# Bone marrow adipocytes provide early sign for progression from MGUS to multiple myeloma

**DOI:** 10.18632/oncotarget.28548

**Published:** 2024-01-16

**Authors:** Bilal M. El-Masri, Benedeta Leka, Fatima Mustapha, Michael Tveden Gundesen, Maja Hinge, Thomas Lund, Thomas L. Andersen, Marta Diaz-delCastillo, Abbas Jafari

**Affiliations:** ^1^Danish Spatial Imaging Consortium (DanSIC); ^2^Department of Clinical Research, Molecular Bone Histology (MBH) Lab, University of Southern Denmark, Odense, Denmark; ^3^Department of Cellular and Molecular Medicine, University of Copenhagen, Copenhagen, Denmark; ^4^Department of Forensic Medicine, Molecular Bone Histology (MBH) Lab, University of Aarhus, Aarhus, Denmark; ^5^Department of Hematology Odense University Hospital, Odense, Denmark; ^6^Department of Hematology, Lillebaelt Hospital, Vejle, Denmark; ^7^Department of Pathology, Odense University Hospital, Odense, Denmark

**Keywords:** multiple myeloma, MGUS, bone marrow adipocyte

## Abstract

Multiple Myeloma (MM) is the second most common hematological malignancy and is characterized by clonal expansion of malignant plasma cells in the bone marrow. In spite of recent advances in the field of MM, the disease has remained incurable. MM is preceded by a premalignant state known as monoclonal gammopathy of undetermined significance (MGUS), with a risk of progression to MM of 1% per year. Establishing a scalable approach that refines the identification of MGUS patients at high risk of progression to MM can transform the clinical management of the disease, improve the patient’s quality of life, and will have significant socioeconomic implications. Here, we provide evidence that changes in the bone marrow adipose tissue (BMAT) provide an early sign for progression from MGUS to MM. We employed AI-assisted histological analysis of unstained bone marrow biopsies from MGUS subjects with or without progression to MM within 10 years (*n* = 24, *n* = 17 respectively). Although the BMAT fraction was not different between the two groups, bone marrow adipocyte (BMAd) density was decreased in MGUS patients who developed MM, compared to non-progressing MGUS patients. Importantly, the distribution profile for BMAd size and roundness was significantly different between the two groups, indicating a shift toward increased BMAd size and roundness in MGUS patients who developed MM. These early changes in the BMAT could serve as valuable early indicators for the transition from MGUS to MM, potentially enabling timely interventions and personalized treatment strategies. Finally, the AI-based approach for histological characterization of unstained bone marrow biopsies is cost-effective and fast, rendering its clinical implementation feasible.

## INTRODUCTION

Multiple Myeloma (MM) is a malignant hematological neoplasm characterized by the uncontrolled proliferation of abnormal plasma cells within the bone marrow [1]. It represents the second most common hematological malignancy, accounting for a significant portion of cancer-related morbidity and 2% of cancer mortality worldwide [1–3]. Despite recent advances in the understanding and treatment of MM, it remains an incurable disease with a substantial impact on patient outcomes and healthcare systems [4].

MM is preceded by a premalignant condition known as monoclonal gammopathy of undetermined significance (MGUS) [5]. MGUS is characterized by the presence of abnormal monoclonal paraprotein in the blood without clinical signs of organ damage, and is often discovered incidentally when blood tests are done for other reasons [1]. MGUS has a prevalence of approximately 1–2% in the general population below 50 years of age, which increases with age reaching up to 3–8% in the population above 80 years [6–9]. While the majority of MGUS cases remain stable, approximately 1% of MGUS patients progress to symptomatic MM each year, presenting a significant clinical challenge in predicting and managing disease progression. The current approach that is used in the clinic for differentiating the stable and progressive myeloma precursor conditions is mainly based on surrogates’ measures of disease burden, such as bone marrow plasma cell percentage and quantity of serum monoclonal protein [10]. However, the utility of this approach is challenged by the diversity of MGUS patients and the fact that the behavior pattern of MGUS does not always correlate with the disease burden [10]. Therefore, development of a scalable, cost-effective approach for accurate risk stratification and identification of MGUS patients at high risk of developing MM could facilitate early detection of the disease progression, allowing for timely interventions and tailored therapeutic strategies before the onset of end-organ damage. This has the potential to revolutionize the clinical management of high-risk MGUS patients and significantly improve patient outcomes, enhance patients’ quality of life, and reduce the socioeconomic burden associated with MM.

Bone marrow adipose tissue (BMAT) is a major component of the bone marrow microenvironment [11–13]. Although traditionally perceived as a passive filler of the bone marrow cavity, BMAT has lately emerged as an active player with dynamic interactions that extend far beyond its previous characterization [12]. Bone marrow adipocytes (BMAds) not only coexist harmoniously alongside hematopoietic and stromal cell populations within the marrow, but also influence their behavior and function. BMAT has been recently implicated in various physiological and pathological processes [14], particularly in relation to hematological, endocrine, and skeletal disorders [15–23]. Obesity, a well-established risk factor for various types of cancers, including MM, is associated with increased bone marrow adiposity, together with altered hematopoiesis and immune regulation [24, 25]. Several studies have reported a correlation between increased bone marrow adiposity and increased risk of MGUS and progression to MM [21, 25]. These findings suggest that BMAds could play a role in the pathogenesis of MM. On the other hand, we have previously shown that BMAT is significantly decreased in non-treated MM patients compared to MGUS patients and healthy individuals [26], suggesting that BMAds could serve as a potential biomarker for MM progression. Therefore, in this study, we examined whether the composition of the BMAT is different in stable and progressing MGUS patients. We employed artificial intelligence (AI)-assisted histological analyses of unstained bone marrow biopsies and found that BMAd density, size, and roundness are significantly different between the two groups and could provide early signs for progression from MGUS to MM.

## RESULTS AND DISCUSSION

**Figure 1:**
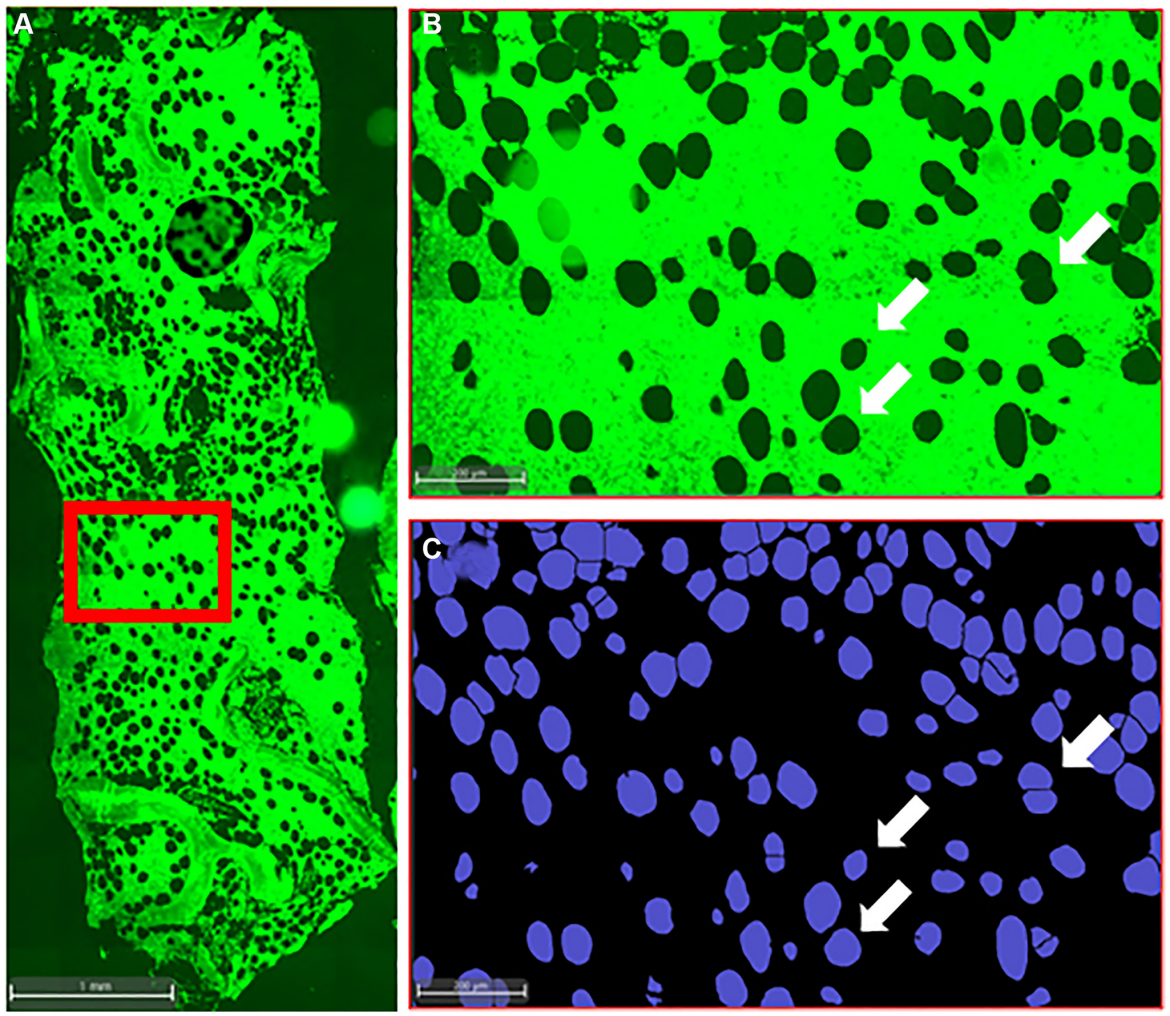
Histological analysis of iliac crest bone biopsy. (**A**) Representative image of the iliac crest bone biopsy from a MGUS patient, illustrating the bone and bone marrow compartments. The autofluorescence highlights the structural arrangement of bone trabeculae and adipose tissue within the bone marrow. (**B**, **C**) Accurate identification of individual BMAds within the bone marrow by AI, indicating the heterogeneity in BMAd size and shape.

**Figure 2:**
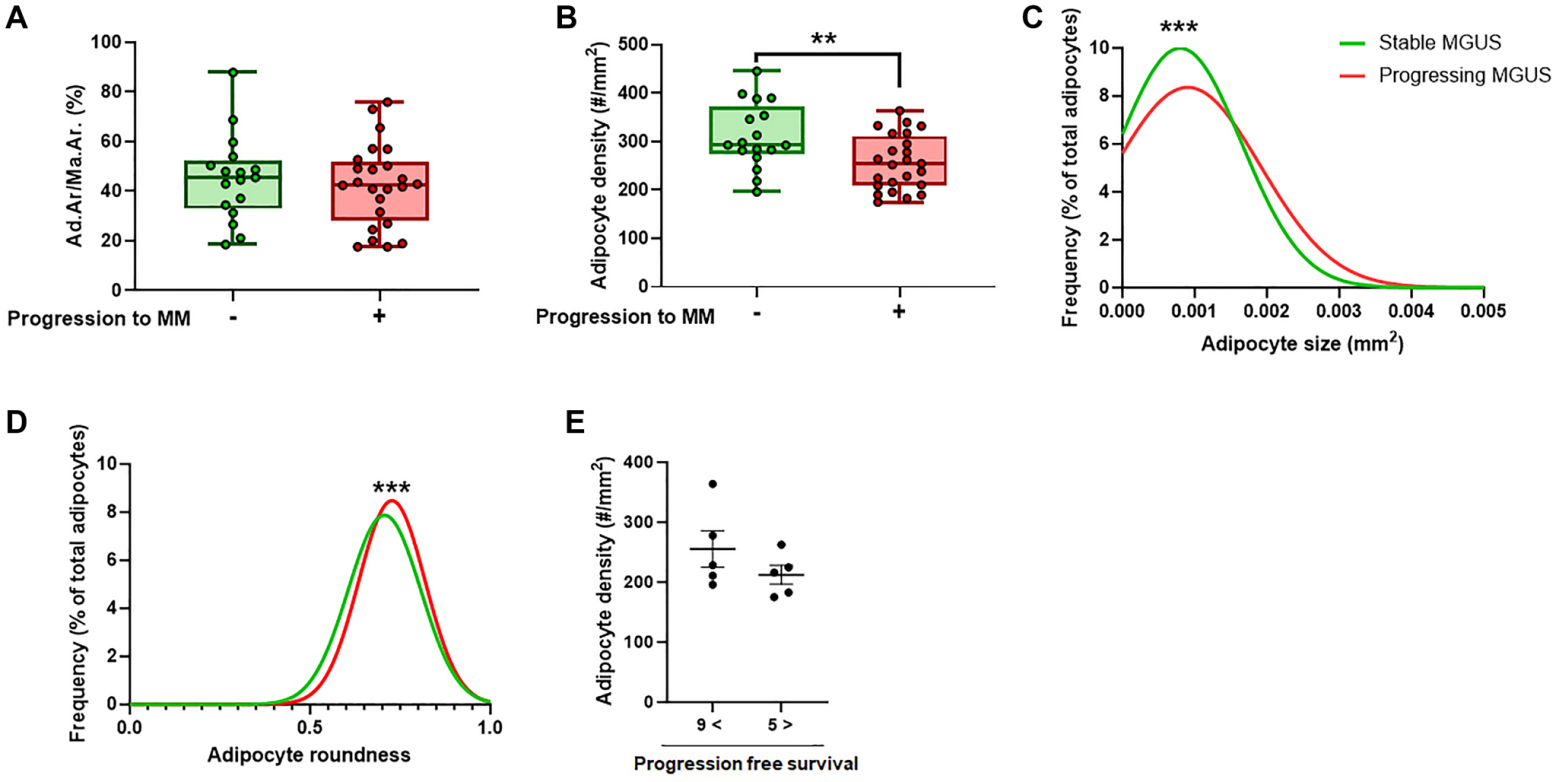
AI-assisted quantitative analysis of BMAT parameters in MGUS subjects with or without progression to MM. (**A**) BMAT fraction within the marrow represented as adipocyte area/marrow area (Ad.Ar/Ma.Ar, %). (**B**) Density of adipocytes within the bone marrow (#/mm^2^). (**C**) Distribution profile of individual BMAd size in stable and progressing MGUS patients. (**D**) Distribution profile of individual BMAd roundness in stable and progressing MGUS patients. (**E**) Density of adipocytes within the bone marrow (#/mm^2^) in low risk MGUS patients who progressed to MM in more than 9 years after obtaining the biopsy compared to MGUS patients who had similar risk stratification score, but progressed to MM in less than 5 years after obtaining the biopsy. Data presented in A–D are obtained from 17 patients with stable MGUS and 24 patients with progressing MGUS. For C and D, number of analyzed BMAds was 26,601 for stable MGUS and 65,394 for progressing MGUS. Error bars on A and B represent standard error of the mean. ^**^ *p* < 0.01, ^****^ *p* < 0.0001. Unpaired Student’s *T*-test was used for A and B, Kolmogorov-Smirnov test was used for C and D.

We employed an AI-assisted approach to perform a systematic and objective histological characterization of the BMAT in progressing and non-progressing MGUS patients, with the aim of identifying possible BMAT features that were different between the two groups. To facilitate translation to high-throughput clinical screening, we utilized unstained bone marrow biopsies by taking advantage of the tissue’s autofluorescence in the FITC channel (Figure 1A–1C). We did not find a significant difference in the BMAT fraction within the bone marrow (% of total marrow) between the stable and progressing MGUS patients (Figure 2A). This suggests that the overall adipose tissue content in the bone marrow was comparable between the two groups. However, we found decreased BMAd density in MGUS patients who experienced progression to MM compared to the non-progressing MGUS patients (Figure 2B). To gain deeper insight into the characteristics of the BMAds associated with MM tumorigenesis, we performed a morphological characterization of the individual BMAds. Importantly, we found a significant shift towards increased BMAd size and roundness in progressing MGUS patients (Figure 2C, 2D). These observations suggest that during MM development, BMAds are subject to alterations which may be indicative of the disease progression. We and others have previously shown that BMAT is significantly reduced in non-treated MM patients [26, 27]. Lack of direct correlation between the bone marrow tumor burden and bone marrow adiposity in overt multiple myeloma, argues against the “space-constricted” mechanism causing decreased BMAT [26]. An alternative proposed mechanism is that cancer cells “hijack” the bone marrow metabolic programs to induce release of fatty acids from BMAds and fulfil their high metabolic demand [28]. It has recently been shown that induction of lipolysis and uptake of fatty acids by MM cells through fatty acid transporter proteins are involved in this process [28]. Therefore, it is possible that the shift towards increased BMAd size in progressing MGUS patients is an early sign that an altered metabolic program induced by MM cells has already started to take effect at this stage, leading to release of fatty acids from BMAds and disappearance of small BMAds, while exerting minor visual impact on the large adipocytes.

As expected, the risk stratification score was notably higher among progressing MGUS patients than their stable MGUS counterparts (Table 1). However, within progressing MGUS patients with comparable risk stratification scores, especially in the low-risk category, we observed a substantial variability in the time taken for progression to MM (progression free survival). Therefore, we aimed at discerning potential differences in BMAT parameters among progressing MGUS patients with similar risk scores but distinct timeframes for progression to MM. Interestingly, we found a discernible trend toward decreased BMAd density in low risk MGUS patients who developed MM within less than 5 years following biopsy collection, as compared to MGUS patients with similar risk score who developed MM in more than 9 years following biopsy collection (Figure 2E). We did not find notable changes in other BMAT parameters between these two groups (data not shown). Additional studies with larger number of patients are required to establish whether decreased BMAd density can be used as a novel risk factor for progression from MGUS to MM.

**Table 1:** Clinical characteristics of the patients

	Stable MGUS (*n* = 17)	Progressing MGUS (*n* = 24)
Age (years; mean ± SD)	42.53 ± 8.96	45.96 ± 11.70
Sex (male *n*, %)	10 (58.82)	6 (25.00)
Serum paraprotein (g/L; mean ± SD)	11.32 ± 8.28	11.62 ± 5.85
Plasma cell burden (%; mean ± SD)	5.04 ± 3.61	4.94 ± 3.22
Risk stratification (low *n*, %)	13 (76.47)	21 (87.50)

As the samples utilized in this study were obtained from biobanks, we did not have access to the data about patients’ weight or ongoing therapies that can affect bone marrow adiposity. Therefore, additional studies are warranted to corroborate the findings presented in this study.

To ensure the validity of AI-generated results, we performed head-to-head comparison between traditional (grid point) and AI-assisted histological analysis of BMAT parameters in a parallel cohort of trephine iliac crest human bone biopsies and observed comparable results (data not show). While one cannot rule out that our trained algorithm incorrectly recognizes empty lumen of blood vessel cross-sections as adipocytes, we estimate these uncommon occurrences to be unlikely and have a dismissible effect among the large adipocyte number detected in each biopsy.

We found a significant difference in the BMAd roundness coefficient between stable and progressing MGUS patients. In addition to the importance of this finding from the perspective of a potential biomarker for early detection of progressing MGUS patients, this finding indicates that the integration of advanced computational analysis and AI-driven algorithms in pathology holds the promise of revolutionizing the field by unraveling hidden patterns and subtle anomalies within the tissues that traditional methods might overlook. This paradigm shift towards computer/AI-augmented pathology could lead to enhanced diagnostic accuracy, personalized treatment strategies, and a deeper understanding of disease mechanisms across a spectrum of medical conditions. The additional advantage of applying this approach on unstained tissue sections highlights its potential for implementation as a cost-effective approach for routine pathological screening.

Taken together, our findings contribute to the growing understanding of the interplay between BMAT and hematological malignancies and the complex role of BMAT in the trajectory from MGUS to MM, urging further exploration and validation of these early indicators of the disease development to improve patient care and outcomes.

## MATERIALS AND METHODS

### Patient cohorts

Iliac crest bone biopsies from 41 MGUS patients were used in this study, including 17 stable MGUS patients who did not progress to MM within 10 years after biopsy collection, and 24 MGUS patients with known progression to MM within 10 years following biopsy collection. The cohort of MGUS patients without progression to MM had a median age of 68 years (55–83 years, ~53% male) and the cohort of MGUS patients with progression to MM had a median age of 69 years (47–83 years, ~29% male). Patient characteristics are presented in Table 1. Patient risk stratification was performed based on myeloma type (IgA or not), M component (<15 g/L or not) and κ/λ ratio (normal or not) as described before [29]. Briefly, patients were stratified into a binary score of “yes” or “no” for each of the described risk factors and the sum of positive outcomes was defined as the overall risk stratification. Patients stratified into risk of progression ≤1 are defined as “low risk of progression”.

### Histological analysis

Formalin-fixed, paraffin-embedded 3-mm trephine iliac crest bone biopsies were sliced into 3.5-μm sections and scanned at 82.6 ms exposure in the FITC immunofluorescent channel of an Olympus VS200 slide scanner. The artificial intelligence (AI) module from HALO (v.3.5, IndicaLabs, NM, USA) was used to detect and characterize bone marrow adipocytes, using the autofluorescence from unstained sections.

### Statistical analysis

Data were analyzed in GraphPad Prism v9.3.1 (GraphPad Inc.) and are presented as mean ± standard error of the mean (SEM) or cumulative histograms. Data were analyzed by unpaired Student’s *T*-test or Kolmogorov-Smirnov test for cumulative distributions.
